# The Use of Chemotherapy to Prolong the Life of Dogs Suffering from Cancer: The Ethical Dilemma

**DOI:** 10.3390/ani9070441

**Published:** 2019-07-14

**Authors:** Tanya Stephens

**Affiliations:** Haberfield Veterinary Hospital, Haberfield, NSW 2045, Australia; tanya7stephens@gmail.com

**Keywords:** chemotherapy, cancer, dogs, oncology, ethical theories, ethical analysis

## Abstract

**Simple Summary:**

Cancer is common in dogs and chemotherapy has advanced significantly in recent years with Veterinary oncology becoming a specialty in many countries such as the UK, Australia and the USA. However there have been no large-scale studies of the potential side effects of chemotherapeutic drugs in companion animals and very little discussion on the ethics of using chemotherapy in these animals. The prognosis for animals suffering from malignant neoplasia (that may be amenable to chemotherapy) generally remains poor and the place of oncology in veterinary medicine can be questioned. Employing the most relevant ethical theories regarding the nature of our duties to animals, the Animal Rights View, the Utilitarian View and the Relational (Contextual) view, this paper examines the ethics of using chemotherapy in dogs with cancer.

**Abstract:**

Despite the emergence some years ago of oncology as a veterinary specialty, there has been very little in the way of ethical debate on the use of chemotherapy in dogs. The purpose of this article is to undertake an ethical analysis to critically examine the use of chemotherapy to prolong the life of dogs suffering from cancer. If dogs have no concept of the future and are likely to suffer at least some adverse effects with such treatments, consideration should be given as to whether it is ethical and in the animal’s best interests to use chemotherapy. Chemotherapeutic drugs are mutagenic, carcinogenic, teratogenic and may be irritant. Furthermore, chemotherapy may involve multiple trips to the veterinarian, multiple procedures and periods in isolation. Cancer-associated pain has been shown to be under-diagnosed and pet owners overestimate the effects of chemotherapy on treatment survival time. Of additional concern is the public health risks associated with chemotherapeutic drugs. As chemotherapy is not generally considered curative, it is in effect palliative care. However, palliative care may not be in the best interests of a terminally ill animal. As the specialty of veterinary oncology continues to grow and as the use of chemotherapy becomes more commonplace in the treatment of animals with cancer, it is imperative that there is an ongoing ethical debate on the use of chemotherapy in animals.

## 1. Introduction

Consideration should be given as to whether it is ethical and appropriate to use chemotherapy to prolong the life of dogs when they have no concept of the future and may suffer adverse effects with such treatment and whether it is in the best interests of the animal.

As animals are increasingly spared from the motor vehicle accidents and infectious diseases of previous years, they are living longer and are increasingly prone to age related diseases such as cancer. Cancer represents one of the major causes of death in dogs accounting for 27% of deaths in purebred dogs in the UK [1]. Cancer accounts for approximately half of the deaths in dogs over the age of ten and approximately one in four dogs will develop cancer during their life [2,3]. In the USA, cancer is the leading cause of death in dogs more than two years of age [4].

Furthermore, as companion animal ownership has increased in economic value, and owners are prepared to spend more on their pets, and companion animals are increasingly seen as “members of the family” [5,6] there may be an expectation by owners that their pets are entitled to the same level of healthcare as human members of the family. At the same time, the development of treatment options and technologies in veterinary medicine has resulted in new ethical dilemmas as to whether a treatment that is available is appropriate for the animal or the owner [7] and overtreatment is an ethical concern given the increasing availability of treatment options and pet insurance, and the emergence of specialties such as oncology [8,9,10]. Fatigue, pain and dyspnea which can be made worse by hypercalcaemia and anaemia, are commonly encountered in animals dying of cancer [11].

Cancer treatments for dogs using chemotherapy have advanced significantly [12,13]. Veterinary oncology has become a specialty in many countries and has been used for over thirty years in some such as the UK and USA. Its introduction to Denmark as a speciality caused debate as there was a concern over the use of a treatment that may prolong life by a few months rather than curing the animal and which has potential significant side effects [14].

That there is a place for oncology in veterinary medicine has been questioned. It has been claimed that “an animal cannot weigh being treated for cancer against the suffering it entails, cannot affirm a desire (or even conceive of a desire) to endure current suffering for the sake of future life” [15]. Ethical issues associated with the use of chemotherapy in cats highlight the ethical concerns about good communication, informed consent, the use of unproven strategies, the ethics of not treating and the ethical issues associated with suboptimal evaluation [16].

Further to this there has been very little discussion on the ethics of using chemotherapy in dogs. Treatment success rates in dogs with cancer is dependent on the type of cancer. Lymphoma, a common cancer in dogs accounting for 8% of cancers is particularly susceptible to chemotherapy. The dog will not be cured by chemotherapy but may have its life prolonged from 2–24 months [12,13].

The dog may feel better, still suffering from the cancer, or it may suffer from the side effects of the treatment. Untreated dogs have an average survival time of 4–6 weeks [12]. Although up to 90% of dogs will go into remission, remission is not a cure (defined as elimination of the disease entirely) so in effect chemotherapy is a form of palliative care and palliation rather than cure, is a major goal of chemotherapy in veterinary oncology [12,13]. Treatment buys time but eventually the majority of dogs will relapse [12,13].

There are of course many types of cancer, with some amenable, and sometimes cured with surgery alone and others which respond to surgery together with other interventions such as chemotherapy and others only amenable to chemotherapy. Chemotherapy is a therapy which is generally not curative and is mutagenic, carcinogenic, teratogenic and involves multiple procedures and periods in isolation.

There are choices that can be made in deciding whether chemotherapy would be appropriate, and these can be made using theories of animal welfare assessment and ethical theories.

## 2. Choices in Dealing with This Dilemma

When confronted with a dog that has cancer that may respond to chemotherapy, if it is not euthanased on diagnosis, there are essentially two choices—either treat with chemotherapy (which is in effect palliative care) or provide palliative care without chemotherapy and euthanase when the animal is suffering. Extension of life with minimal pain and minimal side effects could be argued as good by some.

The act of euthanasia creates ethical issues for the veterinarian [17] and stress which contributes to the “burnout” syndrome in the profession [18]. Stress can be associated with leaving the decision to euthanase too long rather than euthanasing too soon [10,19].

The veterinarian plays an integral role in deciding the course of action and the professional ethics of the veterinarian are central to the decisions that are made. An awareness of the ethical dilemmas that exist as a result of the veterinarian’s unique triangular relationship with the animal and owner, [20,21,22] is important and veterinary decision-making attempts to balance all three interests—those of the animal, the owner and the veterinarian. “In many ways the professional life of veterinarians is more complex and more likely to provide opportunities for conflict than the lives of other professionals” [22].

In addition to balancing the complexity of interests involved in animal care, veterinarians are called upon to assess animal welfare. It is widely recognised that assessments of animal welfare involve a number of assumptions that are ethical in nature [5,23].

In assessing animal welfare in veterinary decision making, it is important to understand theories underpinning animal welfare assessment [5,24]. Several theories may be called upon to provide a framework for determining an appropriate response to the problem of chemotherapy for animals [5].

The Hedonism theory [5] maintains that there should be more pleasure than pain and that a good life is one in which there is a sufficient quantity of positive experiences and sufficiently few negative experiences.

The Preference theory [5] holds that to have a good life it is necessary to achieve what one wants or strives for—that is, to have one’s preferences satisfied.

The Perfectionism theory [5] maintains that a precondition of a good life is to be able to realise significant species-specific potentials.

Answers to the question about what is best for the dog with or without chemotherapy to extend its life, will depend on which of these views is adopted. In addition, quality of life assessments and discussions on when to euthanase in small animal veterinary practice, are useful for the practitioner [25,26,27,28].

Veterinarians are considered to hold Aesculapian authority, uniquely powerful authority, vested in those whom society perceive to be healers and the great power and privilege this gives veterinarians should be used for the benefit of the animal. This authority can be particularly useful in discussions to improve quality of life and directing treatment away from ineffective therapies [20]. In return, society expects a higher degree of integrity and moral behaviour [20]. However, veterinarians need to be aware that their own moral views may differ from those of their clients. To avoid overruling these views, it is important, in any discussion leading to a decision regarding the euthanasia of an animal, to be conscious of and take account of alternative ethical considerations about the value of an animal’s life [29].

The decision to use chemotherapy to extend the life of a dog with cancer involves balancing the risk of adverse events versus benefit. Chemotherapy involves multiple trips to the veterinarian and procedures that may be painful [12]. It is considered unlikely that an animal would choose to suffer and it cannot give consent for the chemotherapy. An animal welfare assessment would ask: is there more pleasure than pain and does the dog have its preferences satisfied? Is the dog able to realise species-specific potential given that it is likely to be confined for therapy and unable to engage with the family in a normal manner because of human health risks as a result of the chemotherapy [30]? Chemotherapeutic agents are generally mutagenic and carcinogenic.

Palliative care is described as treatment that relieves the symptoms of a disease or condition without dealing with the underlying cause, and is commonly used in humans for example to gain extra time to say farewell to a relative [31]. Palliative care of terminally ill companion animals can be likewise understood to give the owners time to come to terms with losing a pet.

Palliative care without chemotherapy does not extend the life of the dog but is a common choice given the costs, commitment and uncertain prognosis with chemotherapy. Although veterinarians, particularly veterinary oncologists, may view cancer as just another disease of animals that can be “treated”, animal owners are likely to view cancer from a different perspective and may view chemotherapy in the light of their own experiences or that of their relatives [32]. In addition, chemotherapy, because it is generally mutagenic and carcinogenic, is a public health risk and owners, veterinarians and veterinary nurses may be rightly concerned about its use in animals [32]. Aftercare of a dog undergoing chemotherapy may place an added burden that an owner should take into consideration [30]. Owners may not be properly informed and “veterinarians and owners often ‘collude’ in misinformation” [16]. People can reach radically different conclusions when judging an animal’s quality of life. “Opinions thus often differ regarding the point at which it becomes kinder to euthanase an animal than not to do so, the point at which it becomes kinder not to undertake a potentially painful therapeutic intervention than to do so” [33].

It is considered that if “animals have no conception of themselves, no self-awareness” that “[t]hey live from instant to instant and do not see themselves as distinct entities with a past and a future, then it is difficult to believe that they would choose any suffering, if they could not imagine a future where the suffering has ended” [34]. It has been argued that if an animal has no concept of death and no explicit desire to stay alive, then the quality of life is more important than the quantity and this perspective is supported by animal welfare legislation [35]. “In the case of animals, however, there is no evidence either empirical or conceptual that they have the capability to weigh future benefits or possibilities against current misery”. To entertain the belief that “my current pain and distress, resulting from the nausea of chemotherapy or some highly invasive surgery will be offset by the possibility of an indefinite amount of future time is taken to be axiomatic of human thinking” [36]. Further “it is equally evident that an animal cannot weigh being treated for cancer against the suffering it entails, cannot affirm a desire (or even conceive of a desire) to endure current suffering for the sake of future life, cannot understand that current suffering may be counterbalanced by future life” [37].

## 3. Why Can the Two Choices Seem Right?

Chemotherapy to extend the life of the dog may seem right as although it is not curative there is a possibility that the dog will go into remission and have an extended quality and quantity of life because treatments for cancer are improving all the time [12,13]. The owner is offered hope and the dog may not be unduly affected by the chemotherapy. The dog may indeed benefit from the extra time and attention lavished on it by the owner and the veterinarian and the veterinarian may derive a great deal of satisfaction from treating the dog and helping the client. It is understood that the dog would be euthanased when terminally suffering.

The alternative choice, to use palliative care without chemotherapy, in the true meaning of the term and euthanase when the animal is suffering, may seem right as whilst accepting that there will be no extension of the dog’s life, the owner and veterinarian may view it as kinder to spare the animal from the possible suffering associated with chemotherapy. The owner is able to spend some time with their pet, whilst coming to terms with losing it and the owner is able to accept the situation. The owner and veterinarian may view chemotherapy as inappropriate for dogs and the financial costs too high for an unlikely outcome. If chemotherapeutic drugs are in short supply for the treatment of humans, they may believe that these drugs should be reserved for human use.

Veterinarians need to be aware that in the decision-making process, their own and the client’s moral views may differ, and in a discussion regarding euthanasia, that the veterinarian ought to acknowledge and give weight to alternative ethical considerations about the value of animal life [29].

## 4. Relating Concerns to Ethical Theories

The most relevant ethical theories, or different kinds of views regarding the nature of our duties to animals, have been selected from the five theories described by Palmer and Sandøe, [38] four of which have been applied to companion animals.

### 4.1. The Animal Rights View

This view maintains that “animals that are sentient and have high level cognitive abilities have rights to life, liberty and respectful treatment. The rights of individuals cannot be overridden in order to benefit others” [26] and we must not do avoidable harm.

This view would claim that the sick animal has a right to be offered available treatments with euthanasia only undertaken if the animal is severely affected. The veterinarian should offer chemotherapy if this is considered better than the alternative (with the aim of extending the animal’s life) and the owner’s interests need not be taken into account. At the same time, chemotherapy, because of the side effects and length of treatment, can be harmful to the animal. Treatment of symptoms as in palliative care are assumed to give the animal a good quality of life as well. So either choice has some justification, especially as the prognosis and extension of life with chemotherapy may vary.

With this view however, there is a concern that the animal’s right to life is at the expense of its welfare and the emotional and financial burden on the owner.

### 4.2. The Utilitarian View

The utilitarian view is a consequentialist approach as it is involves making a cost benefit analysis taking into consideration the animal’s welfare, the owner’s quality of life and the welfare of other affected parties including the veterinarian and the owner’s family. In deciding what to do we must consider the welfare consequences for animals as well as potential benefits for humans, as the interests of animals count as much as the interests of humans. Using this view the veterinarian needs to take into account the interests of the animal as well as the owner and society. It can be difficult to weigh up conflicting interests and to determine the weight each concern should have. In weighing up the options, the utilitarian position is that every agent matters equally. Individual rights can be overridden if the overall benefits are greater for the animal to be euthanased. Therefore, if the owner is happy to euthanase the dog with cancer and buy a new dog, this may be the right solution.

At the same time, euthanasia would also be the right decision if the animal were suffering beyond a level with which the owner can cope. In practice, owners will have an animal euthanased because they cannot cope with the emotional stress of looking after a terminally ill pet. The decision must be the one that delivers the most utility and the least harm.

Regarding the interests of the animal, there needs to be a balance between suffering and alertness. Suffering could be alleviated by drugs such as opioids which may heavily sedate the animal or some alertness could be retained at the cost of less pain relief. The hedonist would opt for pain relief, whereas the perfectionist would favour the latter. Using this view, the decision that is made would take into account the animal, the owner, the veterinarian, the costs of treatment, the effects on others (e.g., the need to keep animals undergoing chemotherapy isolated at times) and that society may perhaps question the use of drugs in dogs in a situation where there could be reduced availability for humans.

The veterinarian needs to balance the interests of the animal and the owner and the utilitarian view describes the unique ethical dimension of the veterinary profession.

### 4.3. The Relational (Contextual) View

The relational view is a group of associated views with the core belief that the relationship between the owner and animal is unique and important. This view holds that domestic animals merit special duties from owners because they are in our care and furthermore our treatment of animals might affect our treatment of fellow humans. Because there is value in close relationships, these relationships should be encouraged.

The human–animal bond is considered so important that it should be maintained, and the veterinarian needs to take into account the bond that the owner has with the animal and use that understanding in deciding on treatment options for the animal. If that bond cannot be maintained, then euthanasia may be acceptable.

This decision may be made easier if the human–animal bond is affected by the loss of emotional attachment because of suffering. At the same time, it may be made more difficult if the owner views caring for the terminally ill animal as emotionally rewarding [14]. Using this view, chemotherapy would be used to hopefully prolong the life of the animal so that the human–animal bond could be maintained for as long as possible. Euthanasia would then be considered if this bond was broken because of animal welfare concerns, the emotional state of the owner dealing with the animal or even the financial burden. The owner may be intransigent to having their suffering animal euthanased. In this situation, the veterinarian may be entitled to invoke animal welfare laws for the benefit of the animal. Legally in Australia, companion animals are classified as a chattel and objects of private property rights. However, legislation for the protection of animals recognises that animals suffer when ill-treated and therefore have intrinsic interests which must be protected regardless of their property status, in other words there is a limitation of personal property rights in order to benefit the interests of animals.

“If society will not accept prolonged suffering in an animal for biomedical reasons (that is reasons that benefit humanity in general) it will surely condemn the owner who keeps a suffering animal alive because he or she cannot bear to let it go” [20]. The fact that chemotherapeutic drugs used on the dog may be in short supply for humans would be of no consequence in this view.

Palliative care without chemotherapy would not be an acceptable choice, as this would remove all hope of a possible long term remission or even “cure”.

## 5. Discussion

Veterinarians should consider whether palliative care is ever in the best interests of a terminally ill animal. If an animal has no concept of death then the quality of life is considered more important than the quantity [8] and this perspective is supported by animal welfare legislation [39].

Concerns that the animal is being kept alive for the owner are not unique to chemotherapy and consideration should be a given to whether veterinarians should initiate a treatment just because they can [5]. However, in balancing the interests of the owner, animal and veterinarian there is the added dimension that chemotherapy involves the use of drugs that are mutagenic, carcinogenic, teratogenic and may be irritant [32], and there has been limited research on treatment decision-making by owners with animals diagnosed with malignancies [31].

Human patients, when considering their own health, overestimate intervention benefit [40] so it is not surprising that pet owners generally overestimate the effects of chemotherapy on treatment survival times with potential false expectations. Importantly, owners can make quality of life assessments on the animal undergoing chemotherapy based on their subjective observations rather than functional tasks, personality expression or changes in behaviour which aligns with what is known about the caregiver placebo effect [41,42].

There are other significant concerns with the use of chemotherapy in dogs. Owners of animals undergoing chemotherapy may be poorly informed and despite the fact that chemotherapy is becoming a more common therapy there have been no large scale studies on the prevalence and incidence of side-effects associated with this treatment [32]. Of additional concern is that the public health risks associated with the use of chemotherapeutic agents may not be given adequate attention in veterinary practice [43]. Importantly, cancer-associated pain has been found to be underdiagnosed in companion animals leading to inadequate pain management and diminished quality of life [4,44].

Decisions about euthanasia can be formulated using an ethical matrix [25] or quality of life assessment tools [10]. These are useful but still require the veterinarian to make individual judgements as a one-size-fits-all solution to euthanasia would risk oversimplifying a highly complex case [27]. Additionally, as welfare assessment is subjective, the idea of a perfect quality of life measure is unrealistic [26] although a checklist to guide decisions is useful for both the veterinarian and owner [45].

Informed consent is essential and pet owners would benefit from a better understanding of the pros and cons of using chemotherapy before treatment is initiated [31] and may not truly understand all the implications of various treatment options [46].

The emergence of veterinary hospice and palliative care as distinct areas of veterinary practice has brought a new dimension to end of life decision making [47,48]. However, there is a lack of scholarly research on this new development to guide clinicians [47] and whilst it has been argued that hospice care is “not giving up too soon” [49] it can also be argued that the establishment of veterinary hospices only panders to “misguided anthropomorphism” [50].

## 6. Conclusions

Despite the emergence some years ago of oncology as a veterinary specialty, there has been very little in the way of ethical debate on the use of chemotherapy in dogs.

Cancer is a common disease in dogs and the decision to treat with chemotherapy raises ethical issues for the owner, society and for veterinarians. There is a need to balance the welfare of the animal, the owner’s interests, the role of the veterinarian and societal expectations. As all veterinarians in companion animal practice are aware, “end of life” decisions can be difficult in regard to “drawing the line”. The three ethical views presented above together with an assessment of animal welfare and clinical decision making by the veterinarian can help to clarify what the issues are, at the same time keeping in mind that legally and ethically the primary responsibility of the veterinarian is to the animal and not the owner [39].

Veterinarians should avoid abrogating their professional decision making onto the owner at the expense of the animal and a good decision regarding a terminally ill animal is important for the well-being of veterinarians, humane outcomes for animals, strengthening the human–animal bond and maintaining the reputation of the profession. Decisions should be based on a knowledge of animal welfare assessment and ethical values, focused on the animal as the first priority of the veterinarian, good clinical skills and good communication can enable veterinarians to make confident decisions when working with pet owners.

Chemotherapy involves using drugs that are mutagenic, carcinogenic, teratogenic and may be irritant, and chemotherapy may involve multiple trips to the veterinarian, multiple procedures and periods in isolation. Cancer-associated pain has been shown to be under-diagnosed and pet owners overestimate the effects of chemotherapy on treatment survival time. As chemotherapy is not generally considered curative then it is in effect palliative care. Palliative care, with or without chemotherapy may not be in the best interests of an animal.

As oncology as a veterinary speciality continues to grow and as the use of chemotherapy becomes more commonplace in the treatment of animals with cancer, it is imperative that there is an ongoing ethical debate and consideration of the ethics of using chemotherapy in animals and whether it is in the animal’s best interests.

## References

[1] Dobson J.M. (2013). Breed-Predispositions to Cancer in Pedigree Dogs. ISRN Vet. Sci..

[2] Bronson R.T. (1982). Variation in age at death of dogs of different sexes and breeds. Am. J. Vet. Res..

[3] Dams V.J., Evans K.M., Sampson J., Wood J.L.N. (2010). Methods and Mortality results of a health survey of purebred dogs in the U.K.. J. Small Anim. Pract..

[4] Fan T.M. (2014). Pain Management in Veterinary Patients with Cancer. Vet. Clin. Small Anim..

[5] Sandøe P., Christiansen S.B. (2008). Ethics of Animal Use.

[6] Walsh F. (2009). Human-animal bonds. Farm Process.

[7] Knesl O., Hart B.L., Fine A.H., Cooper L. (2016). Opportunities for incorporating the human-animal bond in companion animal practice. J. Am. Vet. Med. Assoc..

[8] Sandøe P., Corr S., Palmer C. (2016). Companion Animal Ethics.

[9] McKenzie B.A. (2016). Overdiagnosis. JAVMA.

[10] Knesl O., Hart B.L., Fine A.H., Cooper L., Patterson-Kane E., Houlihan K.E., Anthony R. (2017). Veterinarians and Humane Endings: When is it the right time to euthanize a companion animal?. Front. Vet. Sci..

[11] Gregory N.G. (2004). Physiology and Behaviour of Suffering Animals.

[12] Withrow S.J., Vail D.M., Page R. (2013). Withrow and MacEwen’s Small Animal Oncology.

[13] Childress M.O., Ramos-Vara J.A., Ruple A. (2018). Retrospective analysis factors affecting clinical outcome following CHOP-based chemotherapy in dogs with primary nodal diffuse large B-cell lympoma. Vet. Comp. Oncol..

[14] Kristensen A.T., Sandøe P., Christiansen S.B. (2008). ‘Companion Animals’. Ethics of Animal Use.

[15] Rollin B.E. (2006). Euthanasia and Quality of Life. JAVMA.

[16] Moore A.S. (2011). Managing cats with cancer: An examination of ethical perspectives. J. Feline Med. Surg..

[17] Manette C.S. (2004). A reflection on the ways veterinarians cope with the death, euthanasia and slaughter of animals. J. Am. Vet. Med. Assoc..

[18] Hart L.A., Hart B.L., Mander B. (1990). Humane euthanasia and companion animal death: Caring for the animal, the client and the veterinarian. J. Am. Vet. Med. Assoc..

[19] Batchelor C.E.M., McKeegan D.E.F. (2012). Survey of the frequency and perceived stressfulness of ethical dilemmas encountered in U.K. veterinary practice. Vet. Rec..

[20] Rollin B.E. (2006). An Introduction to Veterinary Medical Ethics, Theory and Cases.

[21] Main D.C.J. (2006). Offering the best to patients: Ethical issues associated with the provision of veterinary services. Vet. Rec..

[22] Brennan A. Competition, Welfare and Ethics in Veterinary Practice. Proceedings of the Australian Veterinary Association Annual Conference.

[23] Tannenbaum J. (1989). Veterinary Ethics.

[24] Sandøe P., Forkman B., Jensen K.K. The interaction of ethical questions and farm animal welfare science. Proceedings of the 2012 RSPCA Australia Scientific Seminar.

[25] Mullan S., Main D. (2001). Principles of ethical decision-making in veterinary practice. Practice.

[26] Yeates J.W., Main D. (2009). Assessment of companion animal quality of life in veterinary practice and research. J. Small Anim. Pract..

[27] Yeates J.W. (2010). Ethical aspects of euthanasia of owned animals. Practice.

[28] Yeates J.W. (2011). Is ‘a life worth living’ a concept worth having?. Anim. Welf..

[29] Sandøe P., Christiansen S.B. (2007). The value of animal life: How should we balance quality against quantity?. Anim. Welf..

[30] Hayes A. (2005). Safe use of anticancer chemotherapy in small animal practice. Practice.

[31] (2016). Collins English Dictionary.

[32] Williams J., Philips C., Byrd H.M. (2017). Factors which influence owners when deciding to use chemotherapy in terminally ill animals. Animals.

[33] Kirkwood J.K. (2007). Introduction—Quality of life: The heart of the matter. Anim. Welf..

[34] Singer P. (2010). Practical Ethics.

[35] McKeegan P. (2011). Quoted in: BVA Congress Report: ‘Where do you draw the line on treatment?’. Vet. Rec..

[36] Rollin B.E. (2007). Ethical issues in geriatric feline medicine. J. Feline Med. Surg..

[37] Rollin B.E. The ethics of small animal cancer treatment. Proceedings of the Michigan State University College of Veterinary Medicine Conference.

[38] Palmer C., Sandøe P., Appleby M.C., Mench J.A., Olsson I.A.S., Hughes B.O. (2011). Chapter 1: Animal Ethics. Animal Welfare.

[39] Stephens T., Bowden P. (2012). Veterinary ethics, Chapter 19. Applied Ethics: Strengthening Ethical Practices.

[40] Hoffmann T.C., Del Mar C. (2015). ‘Patients’ expectations of the benefits and harms of treatments, screening, and tests: A systematic review. Jama Intern. Med..

[41] Conzemius M.G., Evans R.B. (2012). Caregiver placebo effect for dogs with lameness from osteoarthritis. JAVMA.

[42] Gruen M.E., Dorman D.C., Lascelles B.D.X. (2017). Caregiver placebo effect in analgesic clinical trials for cats with naturally occurring degenerative joint disease-associated pain. Vet. Rec..

[43] Edery E.G. (2017). Chemotherapy drug handling in first opinion small animal veterinary practices in the U.K.: Results of a questionnaire survey. Vet. Rec..

[44] Bell A., Helm J., Reid J. (2014). Veterinarians’ attitudes to chronic pain in dogs. Vet. Rec..

[45] Edney A. (1989). Killing with kindness. Vet. Rec..

[46] Christiansen S.B., Kristensen A.T., Lassen J., Sandøe P. (2016). Veterinarians’ role in clients’ decision-making regarding seriously ill companion animal patients. Acta Vet. Scand..

[47] Goldberg K.J. (2016). Veterinary hospice and palliative care: A comprehensive review of the literature. Vet. Rec..

[48] Cooney K.A. (2016). The emerging world of animal hospice: (1) Introduction. Practice.

[49] Cooney K.A. (2016). The emerging world of animal hospice: (2) Management strategies. Practice.

[50] Oakes E. (2016). Hospice and palliative care. Vet. Rec. Lett..

